# Normalised, accessible, and addictive: Socioecological drivers of gutka consumption among urban cloth shopkeepers in Karachi, Pakistan

**DOI:** 10.1371/journal.pgph.0007567

**Published:** 2026-11-05

**Authors:** Sonia Sameen, Rachna Gulrajani, Rahat Batool, Wajiha Anwer, Aiman Khan, Samrah Jawed, Asad Allana

**Affiliations:** 1 Department of Community Health Sciences, Aga Khan University, Karachi, Pakistan; 2 Department of Medicine, Aga Khan University, Karachi, Pakistan; 3 Department of Paediatrics and Child Health, Aga Khan University, Karachi, Pakistan; PLOS: Public Library of Science, UNITED STATES OF AMERICA

## Abstract

Gutka, a commercially prepared smokeless tobacco product, is widely consumed in Pakistan and poses significant public health risks, including oral cancer and other chronic conditions. Cloth shopkeepers represent a particularly vulnerable occupational group due to long working hours, affordability pressures, and high-exposure social environments. This study aimed to examine the multi-level drivers of gutka initiation and sustained use among cloth shopkeepers in Karachi using a socioecological framework and to understand participants’ perceived health impacts and perspectives on existing public health and regulatory interventions. A qualitative exploratory study was conducted with 25 male cloth shopkeepers recruited from five markets across Karachi using quota sampling. In-depth interviews were conducted within participants’ workplace settings. Data were analysed using an inductive-deductive thematic analysis approach guided by the socioecological framework. Ethical approval was obtained from the Aga Khan University Ethics Review Committee. Four overarching thematic categories emerged across 12 sub-themes. At the individual level, participants demonstrated awareness of gutka's health risks, yet continued use through cognitive rationalisation, psychological and physical dependence, early initiation in adolescence, and affordability. At the interpersonal level, peer and family influence during formative years and within the workplace drove initiation and sustained use through normalisation. At the community level, gutka use was deeply embedded in market culture, with widespread social acceptance and unrestricted accessibility through informal supply networks. At the policy and structural level, participants described a clear policy-practice gap, with regulatory bans perceived as ineffective due to systemic corruption, advance raid warnings, and adaptive supply and consumption strategies that circumvented enforcement. Gutka consumption among cloth shopkeepers in Karachi is a complex, multi-determined behaviour sustained by addiction, social normalisation, affordability, and structural failures in regulation. Effective control requires strategies that simultaneously strengthen enforcement, address informal supply networks, provide accessible cessation support, and tackle deep-rooted social norms within occupational settings.

## Introduction

Tobacco use remains a leading cause of preventable morbidity and mortality worldwide, accounting for 8.7 million deaths annually [1,2]. Among its many forms, smokeless tobacco, consumed through chewing, sniffing, or oral placement, is particularly prevalent across South Asia, where it is highly prevalent and socially entrenched within cultural, social, and occupational life [2,3]. More than 300 million people consume smokeless tobacco globally [3], with the highest burden concentrated in India, Bangladesh, and Pakistan [4]. In Pakistan, 8.6% of the population uses smokeless tobacco, with rates of 13.7% among males and 3.9% among females [5]. Commonly used products include paan, gutka, naswar, and chalia, all containing varying combinations of betel nut, areca nut, and tobacco [6]. Regardless of form, smokeless tobacco use is associated with serious chronic disease, including oesophageal, pancreatic, and oral cancers [6–8].

Gutka, a commercially prepared mixture of areca nut, tobacco, slaked lime, and flavouring agents, is among the most widely consumed and harmful smokeless tobacco products in urban Pakistan. Its addictiveness stems from the combined pharmacological effects of nicotine and areca nut, compounded by flavouring agents that enhance palatability and accelerate habituation [9,10]. The rapid onset of effects, including stimulation, appetite suppression, and attenuation of fatigue, distinguishes gutka from other smokeless tobacco products and makes cessation particularly difficult even among users who are fully aware of its harms [10,11]. In Karachi, gutka consumption has been directly linked to a rising burden of oral cancer, now among the most common malignancies in the city [12,13]. A study by Merchant et al. found that chewing betel, areca nut, and tobacco was associated with an 8.5- to 10-fold increase in oral cancer risk after adjusting for covariates [14]. Beyond cancer, gutka use contributes to leukoplakia, erythroplakia, oral submucous fibrosis, gum recession, and tooth staining, imposing a substantial disease burden on urban Pakistani communities [12].

Despite being formally banned in Pakistan, gutka remains widely produced, distributed, and consumed. The ban has not eliminated supply but has instead driven distribution into informal channels, where enforcement has been reported as inconsistent and regulatory actions are frequently anticipated and circumvented by sellers and consumers alike [15]. This policy-practice gap represents a significant challenge in smokeless tobacco control in Pakistan and reflects broader structural difficulties in regulating banned substances within informal urban economies [16].

A particular area of concern within this landscape is gutka consumption among cloth shopkeepers, an occupational group whose work environment may create conditions particularly conducive to sustained use. Cloth shopkeepers are characteristically stationary, occupying fixed premises for working hours that frequently exceed twelve hours, with limited access to food vendors nearby, conditions in which gutka's capacity to suppress hunger, sustain alertness, and attenuate fatigue fulfils a functional role that few affordable alternatives can replicate [17,18]. The physical configuration of cloth markets, with densely clustered adjacent shops and shared communal spaces, generates near-continuous peer exposure, making gutka use not merely accessible but socially unavoidable [19]. Cloth shopkeepers also occupy a distinctive position within the commercial ecosystem where gutka is traded, simultaneously as consumers, as bystanders to enforcement failures, and as participants in the informal market culture that perpetuates its use. Unlike mobile vendors, transport workers, or other retail groups that have received some attention in the South Asian tobacco literature, cloth shopkeepers remain a neglected yet high-risk population [20]. This convergence of occupational vulnerability, social normalisation, affordability, and structural permissiveness forms the rationale for this study's focus.

Qualitative research examining gutka use within occupational settings in Pakistan remains limited. Existing research has predominantly relied on quantitative methods, cross-sectional surveys and prevalence studies that identify the scale of smokeless tobacco use but offer limited insight into the lived experiences, social dynamics, and contextual drivers that sustain it. Studies examining occupational groups have been sparse, and those that exist have focused largely on transport workers and labourers rather than the commercial retail sector. This leaves a gap in the evidence base needed to design interventions that are contextually grounded, occupationally sensitive, and actionable.

### Aim

To address this gap, the present study applies a socioecological framework to explore the multi-level drivers of gutka consumption among cloth shopkeepers in Karachi, aiming to generate a comprehensive understanding of why gutka use persists within this occupational group despite awareness of its harms, and to inform the development of targeted, evidence-based interventions tailored to the conditions of urban informal occupational environments in Pakistan, where unregulated employment conditions and unstructured social environments converge to sustain risk behaviours.

### Objectives

- To explore the individual, interpersonal, community, and structural factors influencing gutka initiation and sustained use among cloth shopkeepers in Karachi.
- To understand the perceived health impacts of gutka use and shopkeepers’ perspectives on the effectiveness of current public health and regulatory interventions.

## Methodology

### Ethics statement

Ethical approval was obtained from the Departmental Review Committee of the Department of Community Health Sciences, followed by the Ethical Review Committee of the Aga Khan University (Approval No: 2025-10790-32996). All participants provided written informed consent after receiving a full explanation of the study's purpose, procedures, and their right to withdraw at any time. Participant confidentiality, privacy, and anonymity were maintained throughout all stages of the study.

### Study design, population and setting

This study employed a qualitative exploratory design to explore the multi-level drivers of gutka consumption among cloth shopkeepers in Karachi, Pakistan, as well as participants’ perceptions of associated health outcomes and the effectiveness of existing public health interventions. The study was underpinned by an interpretivist paradigm, which holds that reality is socially constructed and best understood through the subjective meanings that individuals attach to their experiences and social context. Within this paradigm, a qualitative descriptive approach was adopted, allowing for a comprehensive, contextually grounded account of participants’ experiences and perceptions that remains close to the data without imposing a specific theoretical or philosophical lens such as phenomenology or grounded theory. This approach was selected given the limited evidence on shopkeepers’ lived experiences and the contextual drivers of gutka use, and the need for in-depth, contextually grounded insights that quantitative methods alone cannot provide.

Data were collected from cloth shopkeepers across five markets in Karachi: Hyderi Market in North Nazimabad, Jama Cloth Market in Saddar, Liaquatabad Market in Liaquatabad, Kehkashan Market in Clifton, and Qurtuba Market in Bahadurabad. These markets were selected to capture variation in customer demographics, shop sizes, and socioeconomic profiles, thereby enhancing the diversity and transferability of findings across urban commercial settings.

### Participants and sampling

Participants were recruited using quota sampling, with five shopkeepers selected from each market to ensure representation across diverse socioeconomic backgrounds. Saturation was assessed as meaning saturation, defined as the point at which no new thematic nuances or interpretive insights emerged across the four socioecological levels, rather than the mere absence of new codes. This was tracked iteratively by comparing the coding framework across markets and successive interviews. Saturation was judged to have been reached by approximately the twentieth interview, with the remaining five interviews, required to complete the quota, serving as a confirmatory check that yielded no additional codes or patterns; this was recorded in a saturation log documenting the interview at which each code and theme first appeared. A total of 25 in-depth interviews were conducted. This sample size is consistent with qualitative literature recommending 15–30 participants as sufficient for thematic saturation in in-depth interview studies [21].

To be eligible for inclusion, participants were required to be male cloth shopkeepers aged 18 years or above, currently working in one of the selected markets, and consuming gutka for a minimum of three months. Female participants were not included as sociocultural norms in Pakistan largely preclude women's participation in cloth shopkeeping within designated marketplace settings. While women may engage in similar work independently or from home-based settings, their presence as shopkeepers within formal market spaces such as those studied here is negligible. Gutka, as understood and used by participants, included both commercially manufactured products and homemade preparations made from similar core ingredients (areca nut, tobacco, and slaked lime); both were treated as variants of the same product rather than as distinct substances. Shopkeepers with a history of oral cancer or any other major illness were excluded from the study, as the experience of serious illness typically alters consumption patterns, prompts cessation attempts, and introduces a different relationship with gutka use that would not be comparable to the experiences of current active users.

### Data collection

Recruitment for the study commenced on March 3, 2025, and continued until August 15, 2025. In-depth interviews were conducted with all 25 participants within their respective workplace settings. Each interview lasted approximately 30–45 minutes and was conducted in the participant's preferred local language to facilitate open and comfortable discussion. A semi-structured interview guide was developed prior to data collection, informed by a review of existing literature and validated through expert consultation (S1 Text). All members of the study team received training in qualitative data collection methods prior to fieldwork, with particular emphasis on interview technique, reflexivity, and ethical conduct. Interviews were conducted in a private area within each marketplace to ensure confidentiality and minimise social desirability bias.

### Data analysis

All interviews were audio-recorded and transcribed verbatim. Transcripts were reviewed for accuracy against the original recordings prior to analysis. Data were manually coded by the researcher team with experience in qualitative methods, following the six phases of Braun and Clarke's framework [22] for thematic analysis: familiarisation with the data, generation of initial codes, searching for themes, reviewing themes, defining and naming themes, and producing the final report.

Analysis was guided by an inductive-deductive approach, using the socioecological framework as the overarching deductive structure while remaining open to themes emerging inductively from participants’ accounts. Deductively, the four levels of the framework, individual, interpersonal, community, and policy, were applied as thematic categories. Inductively, transcripts were coded openly and without imposing the predefined structure, allowing additional patterns and meanings to surface directly from the data. This dual approach served to both confirm and extend the deductively derived categories, ensuring that the final thematic structure was theoretically grounded and empirically reflective of the participants’ lived experiences (S2 Text).

To enhance rigour and trustworthiness, two researchers independently coded the data, and any discrepancies were resolved by a third researcher through iterative discussion until consensus was reached.

### Researcher’s reflexivity

The research team comprised public health professionals with backgrounds in epidemiology, environmental and occupational health, and qualitative research. The primary researcher, with prior experience in qualitative methods, led the study design, data collection, analysis, and interpretation. Senior team members with expertise in qualitative and community-based research in Pakistan provided methodological oversight throughout the process. The team's prior engagement in public health research within urban informal settings may have sensitised them to occupational and social determinants of health, including substance use behaviours.

To minimise the influence of researcher positionality on data interpretation, analysis was grounded in participants’ own narratives through the use of verbatim quotations and iterative validation of themes. Regular team discussions were held throughout the analytical process to surface and challenge assumptions. The researchers acknowledge that their familiarity with smokeless tobacco-related health risks may have shaped interpretive decisions, and active steps were taken to ensure that findings remained closely aligned with participants’ accounts rather than with prior expectations.

The study is reported in accordance with the Consolidated Criteria for Reporting Qualitative Research (COREQ) checklist (S3 Text).

## Results

A total of 25 male cloth shopkeepers from two districts of Karachi participated in the study. The majority were from the South district (60%, n = 15), while 40% (n = 10) were from the Central district. The mean age of participants was 40.48 years (SD = 10.62), indicating a predominantly middle-aged sample. The mean age at initiation of gutka use was 24.56 years (SD = 11.85); however, qualitative accounts revealed considerable variation, with a substantial proportion of participants reporting first exposure during adolescence, between the ages of 8 and 16 years, while others reported to have initiated use in early adulthood. On average, participants had been working in their respective markets for 9.83 years (SD = 10.27). Sociodemographic characteristics are summarised in Table 1.

**Table 1:** Sociodemographic characteristics of study participants (n = 25).

Characteristic	Mean / n	SD / %
Age (years)	40.48	10.62
Age at initiation of gutka use (years)	24.56	11.85
Duration of working in the market (years)	9.83	10.27
District 1. Central 2. South	10 15	40% 60%

Participants’ educational backgrounds varied considerably, ranging from no formal schooling to primary-level education, with the highest level of attainment reported being matriculation (secondary schooling, up to grade 10). Reported duration of gutka use ranged from the minimum eligibility threshold of three months to over ten years; however, many participants were unable to recall or unwilling to specify an exact duration, and this characteristic is therefore reported as a range rather than a precise mean or median. Average daily consumption similarly varied widely, from approximately 3–5 packets/sachets per day among lighter users to 20–25 packets/sachets per day among the heaviest users, though several participants were reluctant to disclose precise quantities, which may reflect social desirability concerns around the extent of their use. Most participants reported using a combination of commercially manufactured and homemade gutka depending on convenience, although the majority of purchases were commercially sourced for the sake of ease; homemade preparation was typically resorted to when commercial products were less accessible or affordable.

Through thematic analysis guided by the socioecological framework, four overarching thematic categories emerged, encompassing 12 sub-themes across individual, interpersonal, community, and policy levels. The findings are presented below, organised by each level of the framework.

### Theme 1: Individual-level factors

#### Sub-theme 1.1: Awareness of Gutka ingredients and perceived health consequences.

Participants demonstrated a varied level of awareness regarding the composition of gutka. Most could identify the core ingredients, including areca nut, tobacco, slaked lime, and catechu. However, several participants also held misconceptions, attributing the inclusion of substances such as battery acid, intoxicants, and even chicken blood. One participant described his understanding of gutka ingredients as follows:

*“It contains areca nut, lime, tobacco, catechu… but they also put battery acid in it and other harmful chemicals to make it more addictive.”* (Participant 7, Qurtuba Market)

A recurring perception among participants was that commercially prepared gutka was more harmful than homemade varieties. Several shopkeepers expressed the belief that preparing gutka at home using raw ingredients was a safer alternative, as it allowed them to control the composition and avoid the harmful chemicals purportedly added during commercial production:

*“The gutka you get from the market has all sorts of chemicals mixed in. I make my own at home using just betel nut, tobacco, and slaked lime, at least I know what I am putting in it.”* (Participant 3, Hyderi Market)

When asked about the health consequences of gutka use, shopkeepers readily identified oral cancer as the most severe outcome. Other frequently cited effects included mouth ulcers, restricted mouth opening, gum recession, teeth staining, and a burning sensation when consuming spicy foods. Several participants reported having personally observed cases of oral cancer among fellow shopkeepers and community members, suggesting a high level of perceived severity associated with prolonged gutka use:

*“I have seen people whose mouths have completely closed; they cannot eat, they cannot talk properly. It starts with small wounds in the mouth, then the gums pull back, and eventually it turns into cancer.”* (Participant 12, Liaquatabad Market)

#### Sub-theme 1.2: Rationalisation for personal risk.

Despite acknowledging the harmful effects of gutka, many participants engaged in cognitive rationalisation to minimise their own perceived vulnerability. A predominant belief was that the health consequences of gutka were conditional rather than inevitable, contingent upon factors such as the quantity consumed, the duration of use, or specific consumption practices. Several shopkeepers maintained that maintaining good oral hygiene could mitigate the risks:

*“If you eat it and make sure to wash your mouth properly afterwards, nothing will happen to you. The problem is with people who keep it in their mouth for hours and fall asleep with it.”* (Participant 2, Hyderi Market)

Others attributed illness to factors beyond their control, such as divine will, hereditary predisposition, or inherent physical weakness, thereby externalising the risk:

*“Whatever disease Allah has written in a person's destiny, that is what they will get.”* (Participant 1, Hyderi Market)

Statements reflecting prolonged personal use without perceived consequences further reinforced a sense of invulnerability:

*“I have been using it for over twenty years and I am perfectly fine. If it was really that dangerous, I would have been sick by now. It depends on your body, some people have a weak constitution, and they get affected.”* (Participant 9, Kehkashan Market)

These rationalisations revealed a notable disconnect between awareness of health risks at a general level and the internalisation of personal susceptibility, a pattern that sustained continued consumption despite knowledge of its harms.

#### Sub-theme 1.3: Psychological and physical dependence.

Participants described a range of withdrawal symptoms experienced when they were unable to consume gutka, including sweating, irritability, restlessness, difficulty concentrating, and a perceived drop in blood pressure. These accounts pointed to both physical and psychological dimensions of dependence. Several participants explicitly characterised their gutka use as an addiction, drawing comparisons to substances such as alcohol and narcotics:

*“When I do not have it, I feel restless, my hands start shaking, I cannot focus on anything. It is like my body needs it to function. I know it is an addiction, but I cannot help it.”* (Participant 5, Hyderi Market)

Beyond dependence, participants also cited immediate functional benefits as reasons for continued use. These included perceived mental calmness, heightened alertness, suppression of hunger, and the ability to sustain long working hours, benefits that participants viewed as essential for managing the demands of their occupation:

*“It keeps me alert throughout the day. When I eat it, I feel calm and focused, and I do not feel hungry for hours. Without it, I cannot get through a full day of work.”* (Participant 18, Qurtuba Market)

These accounts suggest that the perceived short-term functional benefits of gutka outweighed participants’ concerns about its long-term health consequences, thereby reinforcing habitual use.

#### Sub-theme 1.4: Early initiation and habituation.

The majority of participants reported initiating gutka use during adolescence, typically between the ages of 8 and 16 years. School years were frequently cited as the period of first exposure, with early experimentation gradually transitioning into habitual use. Participants described how initial curiosity, often stimulated by exposure within family or peer settings, led to regular consumption:

*“I started when I was about fourteen or fifteen years old. At first it was just for fun; my friends were doing it, so I tried it too. Before I knew it, I was consuming two to three packets every day.”* (Participant 14, Jama Cloth Market)

Some participants recounted how a single early exposure was sufficient to establish a lasting pattern of dependence, particularly when facilitated by a close family member:

*“My older brother offered me some when I was just a child. That one time was enough; I got hooked immediately. I have been trying to quit ever since, but I have never been able to stop.”* (Participant 16, Jama Cloth Market)

These narratives highlight the significance of early initiation in shaping long-term consumption patterns and underscore that habitual use often becomes entrenched before individuals are developmentally equipped to fully comprehend the health implications of their behaviour.

#### Sub*-*theme 1.5: Affordability as a driver of consumption.

Affordability emerged as a significant factor influencing gutka consumption patterns. Participants described gutka as a substantially cheaper alternative to other oral tobacco products such as paan, making it an economically attractive option for shopkeepers with limited disposable income:

*“If someone eats ten paans a day at twenty-five rupees each, that comes to two hundred and fifty rupees. But with gutka, you can manage the whole day for just one hundred rupees. That is why people switch to it, it is much cheaper.”* (Participant 8, Qurtuba Market)

When the price of commercially prepared gutka increased, some participants reported switching to even more affordable alternatives or preparing homemade mixtures to sustain their consumption at a lower cost:

*“I usually make it at home now. I buy around fifty rupees worth of betel nut and tobacco, and that lasts me the whole day. It is cheaper than buying packets from the market.”* (Participant 4, Hyderi Market)

A few participants noted that price increases had led to a reduction in the quantity consumed, though complete cessation was rarely reported:

*“Since the price went up to one hundred rupees, most people eat two packets instead of four or five. I have cut down to one. But nobody has actually stopped because of the price.”* (Participant 21, Kehkashan Market)

### Theme 2: Interpersonal-level factors

#### Sub-theme 2.1: Peer and family influence on initiation and sustained use.

Interpersonal relationships emerged as a central factor in both the initiation and continued use of gutka. Participants frequently described how their first exposure to gutka occurred within close social circles, through family members, friends, or co-workers. The influence of older relatives who modelled gutka use during participants’ formative years was particularly salient:

*“When I was a child, my mother used to eat it regularly. She would send me to buy it for her. I did not start then, but I watched her closely. Eventually, when I grew older, I started using it myself.”* (Participant 15, Jama Cloth Market)

Peer influence during adolescence and within the workplace was equally prominent. Several participants described how their initial experimentation was driven by a desire to conform to the behaviour of friends and colleagues:

*“All my friends at school were eating it. I felt curious and thought, let me try it too, it was something new and exciting. After that, it quickly became a firm habit, and I could not stop.”* (Participant 13, Jama Cloth Market)

Participants also highlighted the role of workplace social dynamics in reinforcing the habit and precipitating relapse among those who had attempted to quit. The constant presence of gutka-using colleagues within the market environment was described as a persistent trigger:

*“I tried quitting twice, but when you sit in the shop all day and everyone around you is eating it, you cannot resist. Someone offers it to you, and you take it. The environment pulls you back.”* (Participant 11, Liaquatabad Market)

#### Sub-theme 2.2: Social modelling and normalisation of use.

Closely related to peer influence, participants described a process of social modelling whereby observing the widespread consumption of gutka in their immediate environment gradually normalised the behaviour. Rather than viewing gutka use as a deliberate or conscious choice, many shopkeepers framed it as an almost inevitable outcome of prolonged exposure to a social milieu where consumption was ubiquitous:

*“There is no special reason for starting; it is a matter of what you see around you every day. When everyone around you is eating it, you naturally think, why not try it too? It becomes normal.”* (Participant 10, Liaquatabad Market)

This normalisation through repeated social exposure appeared to lower the perceived threshold for initiation, transforming gutka use from a risky behaviour into a routine social practice embedded within the fabric of daily market life.

### Theme 3: Community-level factors

#### Sub-theme 3.1: Pervasive social norms and cultural acceptance.

Gutka use was described as deeply embedded within the social and cultural fabric of market communities. Participants estimated the prevalence of gutka consumption among fellow shopkeepers to be approximately 70%, reflecting a culture of routine acceptance that rendered its use an unremarkable aspect of daily life. One participant captured this normative quality through a vivid comparison:

*“It is more common than children eating sweets. A child might have a toffee in the morning and another in the evening, but we are consuming gutka constantly throughout the day from morning until we close shop.”* (Participant 1, Hyderi Market)

Participants consistently noted that despite awareness of health risks, the pervasive social acceptance of gutka use within their communities prevented such knowledge from translating into behavioural change. The normalisation of consumption, sustained by high prevalence and cultural endorsement, created an environment in which individual efforts to quit were undermined by collective norms.

#### Sub-theme 3.2: Unrestricted accessibility and informal supply networks.

Despite the existence of regulatory bans, participants uniformly reported that gutka remained widely and easily accessible. Multiple channels of distribution were described: some vendors displayed products openly, while others sold discreetly to known customers. Participants reported that even minors as young as five or six years could obtain gutka without difficulty:

*“It is available everywhere. Even a five- or six-year-old child can buy it if they want. Fifteen is considered a big age—by that time, most boys in the market are already consuming it regularly.”* (Participant 19, Qurtuba Market)

The emergence of homemade gutka production was identified as an alternative supply mechanism that operated entirely outside formal regulatory oversight. Several participants described how homemade preparation had become a means of evading market-level enforcement while ensuring continued availability:

*“Some shops display it openly, hanging the packets in rows for everyone to see. Others keep it hidden underneath the counter and only give it when someone asks specifically. And now many people just make it at home and sell it quietly.”* (Participant 17, Jama Cloth Market)

Participants also highlighted the troubling practice of using children as intermediaries during periods of enforcement. Adults were reported to send children to purchase gutka on their behalf, exploiting the reduced likelihood of children being stopped or questioned:

*“Whenever there is a crackdown, the adults send children to buy it. The child goes to the shop, collects the gutka, and brings it back. This way the adults avoid getting caught.”* (Participant 20, Jama Cloth Market)

#### Sub-theme 3.3: Weak community-level regulation and enforcement.

A prominent theme across participant accounts was the perceived inefficacy of existing enforcement mechanisms at the community level. Shopkeepers described a pattern of temporary and selective enforcement characterised by sporadic police raids that yielded no lasting impact. A significant contributor to this enforcement failure was the widespread practice of bribery, which participants described as a systemic feature of the regulatory landscape:

*“Those who give big bribes to the police display their products openly. Those who give smaller bribes keep it hidden. Everyone pays something. Some police officers even tell the sellers to sell openly and just pay them a good amount.”* (Participant 6, Liaquatabad Market)

Participants further noted that sellers were often forewarned about impending inspections, allowing them sufficient time to conceal their products. This advance warning, reportedly facilitated by informal communication networks between enforcement personnel and sellers, effectively neutralised the deterrent value of regulatory actions:

*“Before any raid happens, word gets around. The shopkeepers quickly hide everything, and by the time the police arrive, there is nothing to find. It is all a show; nothing actually changes.”* (Participant 22, Kehkashan Market)

### Theme 4: Policy and structural-level factors

#### Sub-theme 4.1: The policy-practice gap.

Although participants were generally aware that official bans on gutka production and sale existed, they consistently described a profound disconnect between policy intent and enforcement reality. Regulatory actions were perceived as performative rather than substantive, with crackdowns characterised as brief, inconsistent, and fundamentally undermined by systemic corruption:

*“There is a ban, but it does not mean anything. You can still get gutka as easily as before. The strictness lasts for a few days, and then everything goes back to the way it was.”* (Participant 23, Liaquatabad Market)

Several participants drew attention to the complicity of enforcement authorities in sustaining the very trade they were mandated to suppress. This perception of institutional corruption eroded participants’ confidence in the efficacy and sincerity of public health policy:

*“The same people who come to shut it down are the ones who take the money. They are supposed to enforce the ban, but they are part of the business. Nothing will change until the system itself changes.”* (Participant 24, Jama Cloth Market)

These findings suggest that regulatory bans, in the absence of transparent and sustained enforcement, have inadvertently shifted the distribution of gutka into informal channels rather than reducing overall consumption.

#### Sub-theme 4.2: Market and consumer adaptation under prohibition.

Participants’ accounts revealed that both sellers and consumers had developed adaptive strategies to circumvent regulatory restrictions, resulting in minimal net impact on gutka availability or use. From the supply side, sellers employed a range of tactics to continue operations, including concealing products, selling exclusively to recognised customers, preparing gutka in advance, and paying regular protection fees to authorities:

*“Sometimes there is a brief period of strictness, but even then, gutka is still available if you know where to go. The real crackdowns only happen when shopkeepers stop paying bribes—otherwise, everything continues as usual.”* (Participant 25, Jama Cloth Market)

On the demand side, price increases resulting from regulatory pressure did not appear to lead to cessation. Instead, consumers adjusted by reducing quantities, switching to cheaper alternatives such as naswar, or preparing gutka at home. These adaptive behaviours on both sides of the supply-demand equation demonstrate the limited effectiveness of prohibition-based strategies in the absence of comprehensive, multi-level interventions:

*“People do not stop just because the price goes up. They find other ways—they buy less, or they make it at home, or they switch to something cheaper. The habit does not go away just because it becomes more expensive.”* (Participant 9, Kehkashan Market).

### Participant perspectives on cessation and control strategies

Participants proposed a range of strategies to address gutka consumption, spanning individual, structural, and prevention-oriented approaches. Their recommendations, though primarily concentrated at the individual and policy levels, offered valuable insights into how current users perceive the pathways to reducing gutka consumption within their communities.

#### Individual-level strategies.

The most frequently cited recommendations centred on individual-level behavioural change, including exercising self-control, seeking psychological counselling, and strengthening personal determination. Several participants expressed the belief that cessation was fundamentally a matter of individual willpower:

*“Quitting requires mental control and strong willpower. If a person truly decides to stop, they can do it. But without that inner determination, no amount of external pressure will work.”* (Participant 6, Liaquatabad Market)

*“I believe psychological counselling is the key. People need professional support to understand their addiction and develop the strength to overcome it.”* (Participant 3, Hyderi Market)

#### Supply-side and structural strategies.

A substantial proportion of participants emphasised the need to address the problem at its source by halting the production and distribution of gutka through strict enforcement, factory shutdowns, and sustained regulatory action:

*“If you stop production at the source, meaning they shut down the factories, cut off the supply, then usage will reduce automatically. As long as it is being made, people will find a way to buy it.”* (Participant 21, Kehkashan Market)

However, participants simultaneously expressed scepticism about the feasibility of supply-side interventions in the current governance landscape, citing corruption, temporary enforcement, and the adaptability of informal supply networks as persistent barriers to meaningful change:

*“The government talks about bans, but nothing happens on the ground. Unless there is honest governance and consistent enforcement, all these bans are just words on paper.”* (Participant 24, Jama Cloth Market)

#### Prevention of youth initiation.

Several participants identified the prevention of gutka use among children and adolescents as a critical priority. They drew a clear distinction between current users, whose addiction they viewed as difficult to reverse, and young people who had not yet initiated use and could still be protected through restricted access and early education:

*“Those who are already addicted, you really cannot do much for them. But the children who have not started yet, they should be protected at all costs. Shopkeepers should refuse to sell to children, and there should be strict penalties for those who do.”* (Participant 17, Jama Cloth Market)

A summary of themes, sub-themes, and core findings, are summarised in Table 2 and the socioecological framework applied to gutka consumption among cloth shopkeepers in Karachi is illustrated in Fig 1.

**Table 2:** Summary of themes, sub-themes, core findings of study participants (n = 25).

Level	Sub-theme	Core finding
**Individual** **(Theme 1)**	1.1 Awareness and perceived risk	Oral cancer recognized; harms rationalized away
	1.2 Rationalization of personal risk	Risk externalized via divine will, constitution, hygiene
	1.3 Psychological and physical dependence	Withdrawal symptoms; perceived functional benefits (alertness, appetite suppression)
	1.4 Early initiation and habituation	Onset ages 8–16; single early exposure established lasting dependence
	1.5 Affordability as a driver	Price rises prompt substitution or home preparation, not cessation
**Interpersonal** **(Theme 2)**	2.1 Peer and family influence	Family modelling and workplace peer dynamics drive initiation and relapse
	2.2 Social modelling and normalization	Ubiquitous use lowers initiation threshold; gutka becomes routine social practice
**Community** **(Theme 3)**	3.1 Social norms and cultural acceptance	~70% estimated prevalence; gutka use culturally unremarkable
	3.2 Accessibility and informal supply	Children as young as 5 could purchase; minors used as proxies during enforcement
	3.3 Weak community-level enforcement	Raids anticipated via informal networks; systemic bribery neutralizes deterrence
**Policy** **(Theme 4)**	4.1 Policy-practice gap	Formal bans perceived as performative; institutional corruption erodes trust
	4.2 Market and consumer adaptation	Concealment, home production, and product substitution render prohibition ineffective

**Fig 1:**
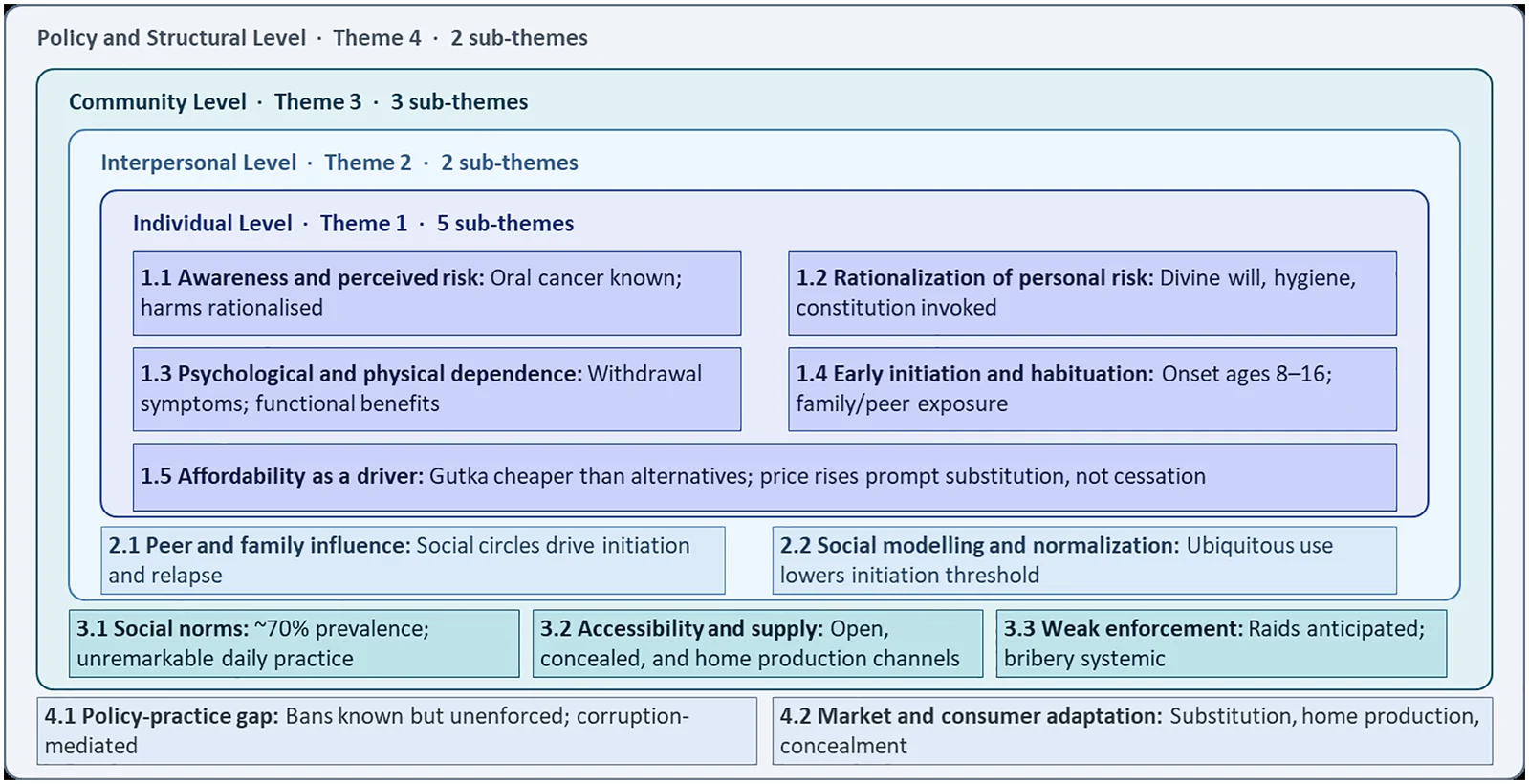
Socioecological framework applied to gutka consumption among cloth shopkeepers in Karachi.

## Discussion

This study provides qualitative evidence that gutka consumption among cloth shopkeepers in Karachi is sustained by a mutually reinforcing set of individual, interpersonal, community, and structural factors, and that the occupational context of shopkeeping creates conditions of particular vulnerability to initiation, habituation, and failed cessation. The central finding is not simply that gutka is addictive and accessible, but that the market environment functions as an ecosystem that normalises, facilitates, and continuously regenerates use, independently of individual awareness of harm. This has important implications for how interventions are designed and targeted within urban informal sector occupational settings.

At the individual level, the findings reveal a consistent and well-documented knowledge-behaviour gap: participants demonstrated clear awareness of gutka's health consequences, particularly its association with oral cancer, yet continued use was sustained by addiction, cognitive rationalisation, and perceived functional utility. This pattern is consistent with evidence from across South Asia, where awareness of smokeless tobacco's harms does not translate into cessation, largely because physiological dependence and the perceived benefits of use, including stress management and sustained alertness, outweigh abstract risk perception [23]. What is distinctive in the present study is the occupational specificity of these functional benefits. Participants did not describe gutka as a general coping mechanism but as an occupational tool, one that enabled them to suppress hunger, sustain concentration, and manage the physical demands of long, unbroken working hours in sedentary market settings. This occupationally embedded functionality represents a unique driver that generic cessation messaging, which addresses health risk without engaging with the working conditions that sustain use, is unlikely to overcome [17].

Early initiation emerged as a critical pathway into long-term dependence, with the majority of participants reporting first exposure between the ages of 8 and 16 years, typically through family members or peers. This is consistent with epidemiological evidence demonstrating that early uptake of smokeless tobacco is a key determinant of long-term use in South Asian populations, and that adolescent initiation is strongly mediated by social modelling within the household and peer group [24,25]. The cognitive rationalisation strategies documented in this study, including attributing risk to divine will, physical constitution, or inadequate oral hygiene, reflect well-established mechanisms of risk minimisation that are particularly resistant to behaviour change interventions focused on information provision alone [26]. Gupta and Cecily similarly documented that among working populations in South Asia, nicotine dependence and the integration of smokeless tobacco into daily occupational routines created a self-reinforcing cycle of use that persisted despite health awareness [27].

Affordability functioned as both a driver of initiation and a barrier to cessation. Participants consistently described gutka as significantly cheaper than alternative oral tobacco products, and price increases did not produce cessation but rather prompted adaptive behaviours including reduced quantity, substitution with cheaper products such as naswar, and home preparation of gutka mixtures. This price inelasticity of demand is well-documented in the smokeless tobacco literature [28]. An analysis by Khan et al. across four South Asian countries found that smokeless tobacco products remain highly affordable relative to income, contributing to continued use and product substitution rather than cessation when prices increase [29]. These findings highlight that price-based regulatory measures, in isolation, are insufficient to reduce consumption among price-sensitive, low-income populations when alternative and informal products remain freely accessible.

At the interpersonal level, family and peer environments shaped both initiation and sustained use across participants’ life courses. Early exposure within households, combined with workplace social dynamics that normalised use and created persistent relapse triggers among those who had attempted to quit, illustrates the cumulative interpersonal pressure operating on this occupational group. These findings align with evidence from South Asia demonstrating that social modelling within family and peer networks is a primary mechanism through which smokeless tobacco use is transmitted across generations and reinforced within shared environments [30,31]. Critically, the workplace itself functioned as an interpersonal risk environment in ways that distinguish shopkeepers from other smokeless tobacco-using populations. Unlike household or community exposure, occupational exposure is near-continuous, structured, and difficult to avoid, as participants described colleagues who offered gutka throughout the working day and environments in which declining constituted a social rupture [32].

At the community level, gutka use was thoroughly integrated into the social fabric of market life, with participants estimating prevalence among fellow shopkeepers at approximately 70%. This high community-level prevalence created a normative environment in which use was rendered routine and individual cessation efforts were consistently undermined by collective norms and constant availability. Similar patterns of social normalisation sustaining smokeless tobacco use despite harm awareness have been documented in Pakistani community settings [31,33]. However, the present study extends this evidence by situating normalisation within a specific occupational context, demonstrating that the market environment amplifies community-level normalisation through the co-location of consumers, suppliers, and a shared occupational identity that incorporates gutka use as a routine social practice.

The findings at the structural and policy level revealed a profound policy-practice gap in which formal regulatory bans on gutka had limited impact on availability or consumption. Enforcement was widely described as performative, undermined by systemic bribery, and effectively pre-empted by informal communication networks that warned sellers of impending raids. Both sellers and consumers had developed adaptive strategies that rendered prohibition largely ineffective, including home production, concealed sale, and the use of children as purchase intermediaries. These findings are consistent with evidence from South Asia documenting significant implementation gaps in smokeless tobacco regulation and the role of interconnected informal distribution systems in sustaining product availability despite formal prohibition [16,34,35]. The present study adds a ground-level account of how these systemic failures are experienced, interpreted, and navigated by the very occupational group that regulation is intended to protect, illustrating that enforcement failure is not merely an institutional shortcoming but a lived reality that erodes trust in public health governance and diminishes the perceived legitimacy of regulatory action [36]. Furthermore, the transition to homemade gutka production constitutes a qualitatively distinct mechanism of regulatory evasion. Unlike commercial manufacture and retail, decentralised household-level production operates below the threshold of regulatory visibility. As the Sindh ban explicitly targets large-scale manufacture and sale, informal home-based production and its distribution through existing social networks remain structurally beyond the reach of conventional enforcement instruments [37]. Participant accounts of purchasing component ingredients, such as areca nut, tobacco, and slaked lime, separately and combining them at home reflect a consumer-level manifestation of a broader circumvention logic documented in South Asian tobacco control literature. This mirrors the commercially organised twin-packet tactic reported in India, whereby manufacturers sell paan masala and loose tobacco as separate legally compliant products, allowing consumers to mix their own gutka at point of purchase [38,39]. Whether enacted at the industrial or individual level, the disaggregation of a banned combined product into its freely available constituent ingredients represents a fundamental structural vulnerability in product-level prohibition, one that cannot be addressed through supply-side enforcement alone.

Product substitution warrants closer attention given its regulatory implications. Participants described switching to naswar, reducing purchased quantities, or shifting to homemade preparation when commercial gutka became less accessible or affordable, but did not frame these as safer alternatives, rather, as functionally equivalent substitutes that preserved the same perceived benefits at lower cost. This is a critical distinction: naswar carries comparable, and in some formulations greater, carcinogenic risk, and homemade preparation removes even the minimal standardisation associated with commercial manufacture. Left unaddressed, this substitution pattern risks being mistaken for harm reduction when it is, in practice, a lateral shift between products of similar risk. Homemade production also poses a distinct regulatory challenge, occurring at the household level with separately legal, unregulated ingredients, placing it outside the scope of manufacturing-focused bans such as the Sindh Prohibition Act and requiring ingredient-level or demand-side approaches instead.

A notable finding was the mismatch between the strategies participants proposed for reducing gutka use and the multi-level drivers their own accounts identified. Participants concentrated their recommendations at the individual level, emphasising willpower and psychological counselling, and at the structural level, emphasising supply-side control and factory shutdowns. They placed limited emphasis on interpersonal or community-based approaches such as peer support, workplace cessation programmes, or community-led awareness initiatives. This pattern is consistent with evidence from South Asia indicating that tobacco users tend to frame cessation as a matter of individual responsibility, even when their own accounts reveal the dominant role of social, occupational, and structural influences [11,23]. The gap between participants’ explanatory accounts and their proposed solutions reflects a broader challenge in tobacco control: interventions must address the determinants that sustain use, not only those that users consciously identify as relevant. In the context of shopkeepers specifically, this suggests the need for occupationally tailored cessation support that engages with workplace social norms, peer dynamics, and the functional role of gutka within the working day, rather than relying on individual-level behaviour change strategies alone [40].

Although presented sequentially by socioecological level, the findings suggest these levels interact rather than operate independently: affordability persists as a driver largely because community-level accessibility guarantees a cheap alternative when prices rise, while workplace norms make cutting back socially costly rather than merely inconvenient. Likewise, weak enforcement at the structural level sustains the informal supply and homemade production that keep gutka accessible at the community level, which in turn reinforces the interpersonal and individual-level normalisation of use. This suggests gutka consumption functions as a single reinforcing system rather than a set of separate, level-specific problems, implying that interventions targeting only one level are unlikely to succeed unless they also disrupt the availability and normalisation mechanisms operating at the others.

This occupational framing also points toward workplace-specific intervention approaches. Market-specific cessation interventions, delivered through trusted market associations or peer-based models that mirror the existing social structure of these workplaces, may succeed where clinic-based approaches, which require leaving one's place of work, would not. Addressing broader occupational stressors, such as limited rest provisions and income insecurity, may also reduce reliance on gutka as a coping mechanism even independent of cessation-focused efforts.

### Strengths and limitations

This study makes several methodological and substantive contributions. It provides in-depth, contextually grounded evidence on gutka consumption within an understudied occupational group in an urban Pakistani setting, filling a gap in the existing literature that has predominantly relied on quantitative, community-level approaches. The application of a socioecological framework enabled a structured yet flexible analysis across multiple levels of influence, and the inclusion of participants from five geographically and socioeconomically diverse markets strengthened the transferability of findings across comparable urban informal work settings. Conducting interviews within participants’ workplace environments, in their preferred local languages, facilitated authentic and contextually rich accounts.

Several limitations should be considered. The sample was restricted to male cloth shopkeepers in selected markets of Karachi, which limits the transferability of findings to other occupational groups, gender identities, or geographic settings. As a qualitative study, the findings are not intended to be statistically generalisable. Nonetheless, structural features of this occupational context, prolonged stationary hours, limited breaks, near-continuous peer exposure, and embeddedness within an informal, weakly regulated commercial environment, are shared by other informal-sector occupations across South Asia, including street vendors, market traders, and retail workers, suggesting the findings may hold analytic, rather than statistical, transferability where similar conditions of accessibility, informal supply, and workplace normalisation are present. This transferability is not automatic, however, as sector-specific factors such as income stability and customer interaction patterns may shift the relative weight of these drivers elsewhere, and would need dedicated research to confirm. Self-reported data may be subject to social desirability and recall bias, particularly regarding sensitive topics such as informal supply networks, bribery, and patterns of regulatory circumvention, and it is possible that participants underreported involvement in or knowledge of these practices. Additionally, information on duration and quantity of gutka use was subject to imprecise recall and, in some cases, reluctance to disclose exact figures; these characteristics are therefore reported as ranges rather than precise estimates, which should be considered when interpreting patterns of use intensity across participants. Income data were not collected in this study, as direct questions about personal or household income were judged to be culturally sensitive and potentially disruptive to rapport in a context where participants were already disclosing detailed information about a socially stigmatised behaviour; income could therefore not be reported as a participant characteristic. Furthermore, the study did not explicitly explore whether participants were also engaged in informal gutka supply, a phenomenon documented in South Asian contexts where enforcement pressure on dedicated tobacco vendors displaces sales to adjacent informal retailers. Participant accounts of stocking and sharing gutka within social networks suggest this warrants direct investigation in future research. The study also did not include perspectives from enforcement authorities, policymakers, or gutka suppliers, which would have provided a more complete picture of the structural and regulatory dynamics described by participants. Future research should address these gaps through multi-stakeholder qualitative approaches and longitudinal designs capable of capturing changes in consumption patterns over time.

## Conclusion

This study demonstrates that gutka consumption among cloth shopkeepers in Karachi is a complex, multi-determined behaviour sustained by the convergence of individual dependence, interpersonal social pressures, community-level normalisation, and systemic failures in regulatory enforcement. Despite widespread awareness of gutka's association with oral cancer and other serious health consequences, continued use is maintained by physiological addiction, perceived occupational utility, affordability, and a market environment in which gutka use is socially entrenched and structurally facilitated. The findings extend existing evidence on smokeless tobacco use in Pakistan by situating consumption within a specific occupational context, demonstrating that the market environment does not merely reflect broader social norms but actively amplifies and regenerates them through dense peer networks, continuous product availability, and the functional integration of gutka into the rhythms of daily working life.

These findings carry important implications for the design of smokeless tobacco interventions in urban Pakistan. Approaches focused exclusively on individual behaviour change or supply-side prohibition have limited potential to reduce consumption in settings where use is occupationally embedded, socially normalised, and structurally sustained. Effective intervention requires strategies that simultaneously engage with the occupational determinants of use, strengthen and transparently enforce existing regulatory frameworks, address informal supply networks, and provide accessible, contextually relevant cessation support tailored to the working conditions of urban informal sector employees. Specifically, these could include workplace-based cessation services embedded within markets, mobile cessation clinics for high-density commercial areas, peer-support programmes built on existing shopkeeper networks, tobacco-free market campaigns run with market associations, enforcement targeted at youth access, and regulation extending to the raw ingredients used in homemade gutka production. Future research should explore the experiences of other occupational groups in similar settings, examine the perspectives of regulatory actors and policymakers, and evaluate the effectiveness of workplace-based cessation interventions in reducing gutka use within Pakistan's informal commercial sector.

## Supporting information

S1 Data Anonymized summary data underlying the findings of this study, including participant-level sociodemographic characteristics and thematic coding notes from in-depth interviews conducted with 25 male cloth shopkeepers across five markets in Karachi, Pakistan. (XLSX)

S1 Text Semi-structured interview guide used to conduct in-depth interviews with study participants, developed following a review of existing literature and validated through expert consultation. (PDF)

S2 Text Thematic analysis summary presenting the coding framework, theme development process, and supporting evidence across the four socioecological levels examined in this study. (PDF)

S3 Text Completed Consolidated Criteria for Reporting Qualitative Research (COREQ) checklist for the study. (DOCX)
