# Supplementary material for: Normalised, accessible, and addictive: Socioecological drivers of gutka consumption among urban cloth shopkeepers in Karachi, Pakistan
Source: PLOS Glob Public Health. 2026 Nov 5;6(11):e0007567. doi: 10.1371/journal.pgph.0007567 (PMC13739151; doi:10.1371/journal.pgph.0007567)
Supplement: Supplementary file 1 [file pgph.0007567.s002.pdf]

Normalised, Accessible, and Addictive: Socioecological Drivers of Gutka Consumption Among Urban Cloth Shopkeepers in Karachi, Pakistan

In-Depth Interview Guide

Date of IDI (dd/mm/yyyy): ____________

Site of IDI: ________________

IDI Start Time: ________

IDI End Time: _________

Moderator Name: __________

Status of IDI

☐ **Complete** ☐ **Incomplete** ☐ **Refused**

Part 1: Knowledge and Perceptions of Harm

What do you know about gutka and paan masala, including their ingredients and health effects?

- o Probe: Have you personally seen anyone experience health problems due to gutka use?
- o Probe: Do you think these health risks apply to you personally? Why or why not?

How do you perceive their impact on health, both personally and in your community?

- o Probe: Are there any perceived benefits of using gutka or paan masala?

Part 2: Patterns of and Reasons for Use

Can you describe how common gutka and paan masala use is among shopkeepers and in your community?

- o Probe: How is gutka use viewed socially among shopkeepers and peers?

What factors do you think influence people, including yourself, to start or continue using these products?

- o Probe: How does the work environment influence use?
- o Probe: Does gutka affect alertness, hunger, stress, or work performance?
- o Probe: Have family members, friends, or co-workers influenced use?
- o Probe: Have you or others ever tried to quit? What challenges were faced?
- o Probe: What happens when someone is unable to consume gutka?

Part 3: Access to Gutka and Paan Masala

How accessible are gutka and paan masala for different age groups in your community?

o Probe: Are children or adolescents able to access these products easily?

What are your observations regarding their sale and marketing?

o Probe: How has the government ban affected availability?
o Probe: Are there ways sellers or consumers continue access despite restrictions?
o Probe: What are your observations regarding enforcement practices?

<u>Part 4: Resources for Cessation and Prevention</u>

What resources or efforts have you seen in your community to prevent or reduce gutka and paan masala use?

o Probe: Do you think current policies or bans are effective? Why or why not?

• What do you think could be done to help people quit or avoid starting?

o Probe: What strategies may help prevent use among children and adolescents?
o Probe: What kind of support would help current users quit?
