# Supplementary material for: Normalised, accessible, and addictive: Socioecological drivers of gutka consumption among urban cloth shopkeepers in Karachi, Pakistan
Source: PLOS Glob Public Health. 2026 Nov 5;6(11):e0007567. doi: 10.1371/journal.pgph.0007567 (PMC13739151; doi:10.1371/journal.pgph.0007567)
Supplement: Supplementary file 2 [file pgph.0007567.s003.pdf]

Normalised, Accessible, and Addictive: Socioecological Drivers of Gutka Consumption Among Urban Cloth Shopkeepers in Karachi, Pakistan

Tabulated Thematic Analysis Summary

Category	Theme
**Personal Level**	1. Knowledge of gutka ingredients and their consequences on health
	2. Justifications for personal risk
	3. Psychological dependence on gutka
	4. Early initiation
**Interpersonal Level**	1. Peer and family influence
	2. Social modeling
**Community Level**	1. Social norms and high prevalence
	2. Easy accessibility and informal supply
	3. Weak community-level regulation & enforcement
**Policy and Structural-Level**	1. Policy-practice gap and weak enforcement
	2. Market and consumer adjustment under prohibition
**Solutions Proposed by Participants**	1. Structural level suggestions<br>• Sub-theme 1: Institutional reform and supply control<br>• Sub-theme 2: Restricting sale of gutka to minors
	2. Individual-level suggestions
