# Supplementary material for: Normalised, accessible, and addictive: Socioecological drivers of gutka consumption among urban cloth shopkeepers in Karachi, Pakistan
Source: PLOS Glob Public Health. 2026 Nov 5;6(11):e0007567. doi: 10.1371/journal.pgph.0007567 (PMC13739151; doi:10.1371/journal.pgph.0007567)
Supplement: Supplementary file 3 [file pgph.0007567.s004.docx]

**Normalised, Accessible, and Addictive: Socioecological Drivers of Gutka Consumption Among Urban Cloth Shopkeepers in Karachi, Pakistan**

COREQ Checklist - Consolidated Criteria for Reporting Qualitative Research

| **No.** | **COREQ Item** | **Response** | **Location in Manuscript** |
| --- | --- | --- | --- |
| **Domain 1: Research Team and Reflexivity** | | | |
| **Personal Characteristics** | | | |
| 1 | Interviewer/facilitator | Yes | Researcher's Reflexivity section — primary researcher led data collection; team comprised public health professionals with qualitative research experience |
| 2 | Credentials | Yes | Researcher's Reflexivity section — team includes public health professionals with backgrounds in epidemiology, environmental and occupational health, and qualitative research |
| 3 | Occupation | Yes | Author affiliations — Department of Community Health Sciences, Medicine, and Paediatrics and Child Health, Aga Khan University |
| 4 | Gender | Not reported | Not applicable to this study |
| 5 | Experience and training | Yes | Researcher's Reflexivity section — all team members received training in qualitative data collection methods prior to fieldwork |
| **Relationship with participants** | | | |
| 6 | Relationship established | Yes | Data Collection section — interviews conducted in participants' preferred local languages within workplace settings to facilitate comfort |
| 7 | Participant knowledge of the interviewer | Yes | Data Collection section — participants were informed of study purpose and researcher roles prior to interview |
| 8 | Interviewer characteristics | Yes | Researcher's Reflexivity section — researcher positionality and potential influence on data interpretation acknowledged and addressed |
| Domain 2: Study Design | | | |
| **Theoretical framework** | | | |
| 9 | Methodological orientation and theory | Yes | Study Design and Data Analysis sections — exploratory descriptive qualitative approach using Braun and Clarke's thematic analysis guided by socioecological framework |
| **Participant selection** | | | |
| 10 | Sampling | Yes | Participants and Sampling section — quota sampling, five participants per market |
| 11 | Method of approach | Yes | Data Collection section — in-person recruitment within market settings |
| 12 | Sample size | Yes | Participants and Sampling section — 25 participants, consistent with qualitative literature recommending 15-30 for thematic saturation |
| 13 | Non-participation | Not reported | Non-participation rates were not formally tracked |
| **Setting** | | | |
| 14 | Setting of data collection | Yes | Data Collection section — interviews conducted within participants' workplace settings in five markets across Karachi |
| 15 | Presence of non-participants | Yes | Data Collection section — interviews conducted in a private area within each marketplace to ensure confidentiality |
| 16 | Description of sample | Yes | Results section — participant characteristics summarised in Table 1 (age, duration of use, market location, district) |
| **Data collection** | | | |
| 17 | Interview guide | Yes | Data Collection section — semi-structured interview guide developed, informed by literature review and validated through expert consultation |
| 18 | Repeat interviews | No | Single interviews only; sufficient for thematic saturation |
| 19 | Audio/visual recording | Yes | Data Analysis section — all interviews audio-recorded |
| 20 | Field notes | Not reported | Field notes were not formally documented |
| 21 | Duration | Yes | Data Collection section — each interview lasted approximately 30-45 minutes |
| 22 | Data saturation | Yes | Participants and Sampling section — sampling continued until thematic saturation was reached |
| 23 | Transcripts returned to participants | No | Transcripts were not returned to participants; rigor ensured through verbatim quotations and multi-researcher analysis |
| Domain 3: Analysis and Findings | | | |
| **Data analysis** | | | |
| 24 | Number of data coders | Yes | Data Analysis section — two researchers independently coded data; a third resolved discrepancies through iterative discussion |
| 25 | Description of the coding tree | Yes | Data Analysis section and Results section — four thematic categories across 12 sub-themes presented in Table 2 |
| 26 | Derivation of themes | Yes | Data Analysis section — inductive-deductive approach using socioecological framework as deductive structure with inductive coding of emerging patterns |
| 27 | Software | Not used | Manual coding conducted by research team |
| 28 | Participant checking | No | Member checking not conducted; rigor maintained through verbatim quotations, multi-researcher coding, and iterative team discussions |
| **Reporting** | | | |
| 29 | Quotations presented | Yes | Results section — verbatim participant quotations presented throughout all themes and sub-themes |
| 30 | Data and findings consistent | Yes | Results and Discussion sections — findings directly grounded in participant accounts with supporting quotations |
| 31 | Clarity of major themes | Yes | Results section — four major themes and 12 sub-themes clearly defined, named, and illustrated with participant quotations |
| 32 | Clarity of minor themes | Yes | Results section — sub-themes presented with narrative analysis and supporting quotations throughout |

*Note: Items marked 'Not reported' or 'No' reflect either methodological decisions made during the study design phase or aspects not applicable to this study design. These are acknowledged as limitations where relevant in the manuscript.*
