# Supplementary material for: Normalised, accessible, and addictive: Socioecological drivers of gutka consumption among urban cloth shopkeepers in Karachi, Pakistan
Source: PLOS Glob Public Health. 2026 Nov 5;6(11):e0007567. doi: 10.1371/journal.pgph.0007567 (PMC13739151; doi:10.1371/journal.pgph.0007567)
Supplement: Supplementary file 4 [file pgph.0007567.s005.docx]

**Normalised, Accessible, and Addictive: Socioecological Drivers of Gutka Consumption Among Urban Cloth Shopkeepers in Karachi, Pakistan**

Response Sheet

| **Comment** | **Response** |
| --- | --- |
| **Reviewer 1** | |
| The paper uses standard scientific methods, and the paper is robust methodologically. It is relatively weak on the novelty front. I have a few suggestions.  1. Gutka and smokeless tobacco: Gutka is a specific type of smokeless tobacco (tobacco, lime, areca nut and other ingredients). The authors acknowledge this. However, while presenting the quotations from the participants, there seems to be an overlap between smokeless tobacco and Gutka. This is important from policy perspective as Gutka may have been banned at the provincial level (or possibly at the federal level in Pakistan) but raw tobacco may not be covered by the ban. Authors may clarify the ban status of various smokeless tobacco products. The authors may also clarify about the overlap between the two. As pointed out in the data, users shift from one product to another product based on affordability, availability, experimentation, etc. Since the paper is about Gutka, it is important to be elaborate on this. | We thank the reviewer for this important observation. We have clarified in the Methods (Participants and Sampling) that gutka, as used and understood by participants, includes both commercially manufactured products and homemade preparations made from similar core ingredients (areca nut, tobacco, slaked lime); both were treated as variants of the same product. The eligibility criterion and the relevant Table 1 variable have been revised accordingly to refer specifically to gutka use rather than smokeless tobacco use generally. We have also added a sentence to the Discussion noting that the shift toward homemade production represents a structurally distinct mode of regulatory evasion from commercial-scale circumvention, since Sindh's ban targets manufacture and sale at scale, and dispersed household-level production is far harder to detect and enforce against. We believe this strengthens the paper's central policy argument: the ban altered the supply landscape rather than reducing demand or use. |
| 2. Shopkeepers as users or as suppliers: In South Asia, there is anecdotal evidence that tobacco vendors, if targeted by law enforcement agencies, the selling points shift to other shops including cloth shops, which makes law enforcement extremely difficult. It is not clear from the paper whether the participants were also selling tobacco. | We thank the reviewer for raising this important point. While participants were recruited and characterized as consumers, some accounts did describe informal stocking and sharing of gutka within workplace social networks, suggesting the boundary between consumer and informal supplier may not always be clear-cut in this setting. The dual role of cloth shopkeepers as potential informal gutka suppliers was not explicitly explored in the present study, and we have acknowledged this as a limitation. We agree that these warrant direct investigation in future research, particularly given the documented displacement of tobacco sales to non-specialist retail outlets following enforcement pressure on dedicated vendors. |
| 3. In South Asia, there have been manufacturing industries which sell paan masala without tobacco. By this tactic, they are able to legally sell the product and the point-of-purchase, these are often sold along with loose tobacco. Were such dynamics also reported by the participants. If not, is there any evidence in this regard from the preexisting literature? | We thank the reviewer for raising this important dynamic. While the specific commercial practice of selling paan masala without tobacco alongside loose tobacco at point of purchase was not explicitly reported by participants, our data contains evidence of a functionally analogous consumer-level practice. Participants 3 and 4 described purchasing the constituent ingredients of gutka, such as areca nut, tobacco, and slaked lime, separately and preparing homemade mixtures, reflecting the same underlying circumvention logic: when the combined product is banned, its unregulated components remain accessible and can be reassembled by the consumer. We have added a passage to the Discussion connecting this participant-reported behaviour to the twin-packet tactic documented in South Asian tobacco control literature, arguing that whether this disaggregation is commercially organised or individually enacted, it represents a structural vulnerability in product-level prohibition that warrants direct policy attention. |
| 4. Choice of cloth shopkeepers: The authors have used therms such as commercial retail, informal occupational environments but have selected only cloth shopkeepers. What are the underlying reasons? Why did the authors not select any other retailers from Karachi markets? What do the authors mean by informal occupational environment (informal sector or informal environment which exists in every occupational/organisational setting irrespective of whether the setting is formal or informal). | We thank the reviewer for this observation. Cloth shopkeepers were selected over other retail groups because their occupational characteristics create conditions of particular vulnerability to gutka use: they are stationary, work shifts frequently exceeding twelve hours, and have limited access to food nearby, conditions in which gutka's functional properties (hunger suppression, alertness, fatigue management) are acutely relevant. The dense physical configuration of cloth markets also generates near-continuous peer exposure, intensifying social normalization of use. Unlike mobile vendors or transport workers, who have received some attention in the South Asian tobacco literature, cloth shopkeepers represent a neglected yet high-risk group. We have revised the Introduction to make this rationale explicit.  Regarding 'informal occupational environment', we acknowledge this was used ambiguously. We have revised the manuscript to distinguish between the informal sector dimension, referring to the unregistered, unregulated nature of participants' employment, and the informal social environment, referring to the unstructured social atmosphere of market life. Both are relevant and now stated explicitly. |
| 5. Lines 167-169:The authors report having excluded persons with history of cancer and other major illnesses. I can understand the rationale as their views are likely to be far different than the current users. However, I do not agree with the statement that this group will have bias in reporting health outcomes. They already have encountered a Non-communicable disease and I feel that they would simply report it and may (or may not) attribute it to tobacco use but will not have bias in reporting the outcome. Please re-frame the sentence or clarify. | We thank the reviewer for this important clarification. We agree that the original wording was imprecise because individuals with a history of serious illness would not necessarily introduce bias in reporting health outcomes; rather, their experience of gutka use would be qualitatively different from that of current active users, as serious illness typically alters consumption behavior and prompts cessation attempts. The exclusion criterion has been reframed accordingly in the Methods section. |
| 6. Table reflects the quotations presented except 1.2 where divine will has been mentioned. I could not find quotation indicating that. | We thank the reviewer for this careful reading. We agree that the original text did not include a supporting quotation for the divine will sub-theme. A verbatim quotation from the same participant has now been added to the manuscript, in which the participant explicitly attributes illness to divine decree. |
| **Reviewer 2** | |
| This manuscript presents the results of original research in a well-structured and well written manner.  Aim/Objectives: The aim and objectives need to be reviewed and fine-tuned, with the objectives leading to the aim. | We thank the reviewer for this observation. The aim has been clearly labelled as a standalone statement in the Introduction, distinct from the objectives. The objectives have been revised to map explicitly onto the study's socioecological framework — Objective 1 addresses the multi-level drivers examined across the four thematic categories, and Objective 2 addresses participants' health perceptions and intervention perspectives. Together, both objectives now lead clearly into the overarching aim. The abstract has also been updated to reflect this revised wording. |
| Methods: Which approach was used for this qualitative work? Phenomenology, Grounded theory or any other? Please provide details of how the sample size of 25 male cloth shopkeepers was constituted. Exclusion of female participants need to be justified. There is need for data analysis to be undertaken by two independent persons. It is noted that the research work received ethical clearance It is also noted that authors included a paragraph on reflexivity. On the other hand, authors need to adhere to the relevant guidelines for qualitative studies. | The qualitative approach employed in this study is thematic analysis, following the six-phase framework of Braun and Clarke. As this study sought to systematically explore and describe multi-level drivers of gutka use within the established socioecological framework, thematic analysis was the most appropriate and sufficient method, as detailed in the Data Analysis section.  The sample size of 25 was constituted through quota sampling, with five participants selected from each of the five markets to ensure socioeconomic and geographic diversity. This sample size is consistent with qualitative literature recommending 15–30 participants as sufficient for thematic saturation in in-depth interview studies, and saturation was confirmed empirically as no new themes emerged beyond this point. A clarifying sentence has been added to the Participants and Sampling section.  The exclusion of female participants reflects the occupational and sociocultural reality of the study setting rather than an arbitrary methodological decision. In Pakistan, sociocultural norms largely preclude women's participation in cloth shopkeeping within designated marketplace settings. While women may engage in similar commercial activity independently or from home-based contexts, their presence as shopkeepers within formal market spaces including the five markets studied here is negligible. This study therefore focused exclusively on male cloth shopkeepers as they constitute virtually the entire population of cloth shopkeepers in these settings. This justification has now been made explicit in the Participants and Sampling section.  We wish to clarify that data analysis was not undertaken by a single researcher. As reflected in the Author Contributions section, three authors, namely Sonia Sameen, Rachna Gulrajani, and Wajiha Anwer, were involved in the analytical process. Two researchers independently undertook primary coding (Rachna Gulrajani and Wajiha Anwer), with a third (Sonia Sameen) resolving any discrepancies through iterative discussion until consensus was reached. The Data Analysis section has been revised.  The study was conducted and reported in accordance with the Consolidated Criteria for Reporting Qualitative Research (COREQ). Key COREQ criteria are addressed throughout the manuscript: ethical approval is documented in the Ethical Approval section; researcher reflexivity is addressed in the dedicated Reflexivity section; participant characteristics including age, duration of use, and market location are summarised in Table 1; interview setting, duration, and conduct are described in the Data Collection section; audio recording and verbatim transcription are detailed in the Data Analysis section; the analytical framework is named and described with reference to Braun and Clarke; thematic saturation is documented in the Participants and Sampling section; and direct participant quotations are used throughout the Results to substantiate all themes and sub-themes. A completed COREQ checklist has been provided as a supplementary document for the reviewer's reference. |
| Results: There is need to remove the name of the market where the participant is based so as to preserve the anonymity of participants and avoid unnecessary identification.  Conclusions are derived from the data. | We respectfully disagree with this suggestion. The five markets selected for this study: Hyderi Market (North Nazimabad), Jama Cloth Market (Saddar), Liaquatabad Market (Liaquatabad), Kehkashan Market (Clifton), and Qurtuba Market (Bahadurabad), were deliberately chosen to represent the socioeconomic, demographic, and ethnic diversity of Karachi's informal commercial landscape. The study's quota sampling strategy was designed around these five markets to ensure proportional representation across geographically and socioeconomically distinct urban settings. Removing market-level attribution from participant quotations would directly undermine this sampling rationale and strip the findings of their contextual grounding. Karachi is a large and highly heterogeneous metropolitan city and retaining market-level attribution allows readers to contextualise findings within distinct urban settings and assess transferability across comparable contexts. Participant anonymity is preserved through the use of participant numbers rather than names or any other identification. The market name alone does not render any participant identifiable, as each market employs large numbers of cloth shopkeepers. We therefore believe that retaining market attributions strengthens rather than compromises the rigour and contextual richness of the study. |
