# Supplementary material for: Normalised, accessible, and addictive: Socioecological drivers of gutka consumption among urban cloth shopkeepers in Karachi, Pakistan
Source: PLOS Glob Public Health. 2026 Nov 5;6(11):e0007567. doi: 10.1371/journal.pgph.0007567 (PMC13739151; doi:10.1371/journal.pgph.0007567)
Supplement: Supplementary file 5 [file pgph.0007567.s006.docx]

# **Normalised, Accessible, and Addictive: Socioecological Drivers of Gutka Consumption Among Urban Cloth Shopkeepers in Karachi, Pakistan**

**Response Sheet**

| **Comment** | **Response** |
| --- | --- |
| Major Suggestions  1. Enhance Methodological Transparency  While the study is described as a qualitative exploratory design using thematic analysis, the specific qualitative orientation remains unclear. Explicitly stating the epistemological stance and methodological approach (e.g., interpretivist paradigm and qualitative descriptive approach) would strengthen methodological rigor and improve alignment with qualitative reporting standards. | We thank the reviewer for this suggestion. We have revised the Study Design subsection to explicitly state the study's epistemological stance and methodological approach. The manuscript now specifies that the study was underpinned by an interpretivist paradigm and employed a qualitative descriptive approach and clarifies why this approach was chosen over an approach that imposes a specific theoretical lens such as phenomenology or grounded theory. (Page 5, Lines 166–179) |
| 2. Provide a More Detailed Account of Data Saturation  The manuscript indicates that thematic saturation was achieved but does not explain how saturation was determined. Clarification regarding the type of saturation reached (code or meaning saturation), the stage at which it occurred, and the process used to document it would improve methodological credibility. | We thank the reviewer for this comment. We have expanded the Participants and Sampling subsection to clarify that meaning saturation, rather than mere code saturation, was the standard applied. We now specify that saturation was judged to have been reached by approximately the twentieth interview, with the remaining five interviews serving as a confirmatory check, and that this process was documented in a saturation log tracking when each code and theme first appeared. (Page 6, Lines 187–198) |
| 3. Expand Participant Characteristics  The participant profile is currently limited. Inclusion of additional contextual characteristics such as educational status, income category, duration of gutka use, average daily consumption, and type of gutka consumed (commercial versus homemade) would facilitate richer interpretation of the findings. | We thank the reviewer for this suggestion. We have expanded the Results to include participants’ education, type of gutka consumed, and duration and amount of use, reported as ranges. Income data were not collected because asking about income could have affected participant rapport, particularly as participants were discussing a stigmatised behaviour. We have now noted this as a limitation. (Page 8, Lines 273–286; Page 24, Lines 752–760) |
| 4. Strengthen Integration of the Socioecological Framework  The framework is effectively applied in presenting findings; however, the discussion could better articulate interactions across levels. Greater emphasis on how affordability, workplace culture, social norms, accessibility, and policy failures interact to sustain gutka use would enhance the theoretical contribution of the study. | We thank the reviewer for this valuable suggestion. We have added a new paragraph to the Discussion explicitly addressing how the four socioecological levels interact rather than operate independently, illustrating how affordability, workplace norms, community-level accessibility, and structural enforcement failures reinforce one another to sustain gutka use as a single self-reinforcing system rather than a set of discrete, level-specific problems. (Page 22, Lines 707–717) |
| 5. Further Emphasize Occupational Health Implications  One of the manuscript’s most novel contributions is its focus on an occupationally vulnerable population. The discussion would benefit from deeper consideration of workplace-based tobacco control strategies, occupational stress, health promotion programs, and market-specific cessation interventions. | We thank the reviewer for highlighting this. We have added a new paragraph to the Discussion situating gutka use within an occupational health framework, addressing workplace-based cessation strategies, the role of occupational stressors such as prolonged standing and lack of structured breaks, and the potential of market-specific, peer-based cessation interventions delivered through trusted market associations. (Page 19, Lines 583–597; Page 22, Lines 718–724) |
| 6. Expand Discussion on Product Substitution  Participants reported shifting to naswar and homemade preparations when commercial gutka became less accessible or more expensive. This finding has important regulatory implications and warrants a more focused discussion on product substitution, harm reduction misconceptions, and challenges associated with regulating homemade products. | We thank the reviewer for this comment. We have substantially expanded the Discussion to address product substitution in greater depth, including the three substitution pathways participants described, an explicit discussion of why this does not represent harm reduction given naswar's comparable carcinogenic risk, and the distinct regulatory challenge posed by homemade production falling outside the scope of manufacturing-focused bans such as the Sindh Prohibition Act. (Page 21–22, Lines 677–689) |
| Minor Suggestions  7. Refine Aim and Objectives  The distinction between the study aim and objectives could be improved to ensure clearer alignment and reduce overlap. Objectives should directly reflect the socioecological determinants, perceived health effects, and views on existing control measures. | We thank the reviewer for this comment. We have revised the Aim to remove language that duplicated content already captured in Objective 1, thereby clarifying the distinction between the two. The Objectives continue to reflect the socioecological determinants of gutka use, participants' perceived health effects, and their views on existing control measures. (Page 5, Lines 142–157) |
| 8. Strengthen the Conceptual Figure  The current socioecological framework is informative but primarily descriptive. A more explanatory model showing pathways such as early initiation leading to addiction, peer influence fostering normalization, and enforcement failures sustaining availability would enhance the manuscript’s conceptual contribution. | We thank the reviewer for this suggestion. We retained the existing socioecological figure to preserve the framework used to organise the qualitative findings. To strengthen its explanatory contribution, we have expanded the Discussion to explicitly describe the pathways and interactions across levels. The revised text explains how early exposure and peer influence contribute to normalization and dependence, how affordability and accessibility sustain consumption, and how weak enforcement facilitates continued availability through informal supply networks. We further clarify that these factors operate as an interconnected system rather than as independent influences. (Page 22, Lines 707–717) |
| 9. Discuss Transferability  The limitations section could better address the applicability of findings to other occupational groups such as vendors, retail workers, informal-sector employees, and market traders in similar South Asian contexts. | We thank the reviewer for this comment. We have added text to the Limitations section discussing the analytic transferability of our findings to other informal-sector occupations sharing similar structural features, such as street vendors, market traders, and retail workers, while noting that sector-specific factors may shift the relative weight of these drivers elsewhere. (Page 24, Lines 736–748) |
| 10. Provide More Specific Practice and Policy Recommendations  The conclusion would be strengthened by outlining actionable recommendations, such as workplace-based cessation services, mobile tobacco cessation clinics, peer-support programs, tobacco-free market campaigns, enhanced enforcement against youth access, and regulation of ingredients used in homemade gutka production. | We thank the reviewer for this suggestion. We have added specific, actionable recommendations to the Conclusion, including workplace-based cessation services embedded within markets, mobile cessation clinics, peer-support programmes, tobacco-free market campaigns run with market associations, enforcement targeted at youth access, and regulation extending to raw ingredients used in homemade production. (Page 25, Lines 783–798) |
