# Supplementary material for: Normalised, accessible, and addictive: Socioecological drivers of gutka consumption among urban cloth shopkeepers in Karachi, Pakistan
Source: PLOS Glob Public Health. 2026 Nov 5;6(11):e0007567. doi: 10.1371/journal.pgph.0007567 (PMC13739151; doi:10.1371/journal.pgph.0007567)
Supplement: Peer review history

# Peer review: Decision Letter 0

**DOI:** 10.1371/journal.pgph.0007567.r001

**Authors:** Katherine Demi Kokkinias

29 Jun 2026

PGPH-D-26-01732

Normalised, Accessible, and Addictive: Socioecological Drivers of Gutka Consumption Among Urban Cloth Shopkeepers in Karachi, Pakistan

PLOS Global Public Health

Dear Dr. Sameen,

Thank you for submitting your manuscript to PLOS Global Public Health. After careful consideration, we feel that it has merit but does not fully meet PLOS Global Public Health’s publication criteria as it currently stands. Therefore, we invite you to submit a revised version of the manuscript that addresses the points raised during the review process.

The manuscript has been evaluated by two reviewers, and their comments are available below.

The reviewers raised a number of concerns that need attention. They would like additional discussion and distinction made between Gutka and smokeless tobacco. They would like additional discussion on whether the shopkeepers were also the suppliers of Gutka. The reviewers would also like further justification for exclusion of participants with cancer and other major illnesses and more information about the choice of examining cloth shopkeepers. The reviewers would like justification for the exclusion of female participants and how sample size was determined. They have requested that the specific name of the market be removed to improve participant anonymity. Please also provide a copy of the interview guide.

Could you please revise the manuscript to carefully address the concerns raised?

Please submit your revised manuscript by Aug 25 2026 11:59PM. If you will need more time than this to complete your revisions, please reply to this message or contact the journal office at globalpubhealth@plos.org. When you're ready to submit your revision, log on to https://www.editorialmanager.com/pgph/ and select the 'Submissions Needing Revision' folder to locate your manuscript file.

Please include the following items when submitting your revised manuscript:

- A letter that responds to each point raised by the editor and reviewer(s). You should upload this letter as a separate file labeled 'Response to Reviewers'.
- A marked-up copy of your manuscript that highlights changes made to the original version. You should upload this as a separate file labeled 'Revised Manuscript with Track Changes'.
- An unmarked version of your revised paper without tracked changes. You should upload this as a separate file labeled 'Manuscript'.

Guidelines for resubmitting your figure files are available below the reviewer comments at the end of this letter.

As the corresponding author, your ORCID iD is verified in the submission system and will appear in the published article. PLOS supports the use of ORCID, and we encourage all coauthors to register for an ORCID iD and use it as well. Please encourage your coauthors to verify their ORCID iD within the submission system before final acceptance, as unverified ORCID iDs will not appear in the published article. *Only*  the individual author can complete the verification step; PLOS staff *cannot*  verify ORCID iDs on behalf of authors.

We look forward to receiving your revised manuscript.

Kind regards,

Katherine Demi Kokkinias, Ph.D.

Staff Editor

PLOS Global Public Health

Journal Requirements:

1. Please ensure that your Ethics Statement is available in its entirety at the beginning of your Methods section, under a subheading 'Ethics Statement'.

2. Please provide separate figure files in .tif or .eps format.

For more information about figure files please see our guidelines:  LINK

https://journals.plos.org/globalpublichealth/s/figures

https://journals.plos.org/globalpublichealth/s/figures#loc-file-requirements

3. We have noticed that you have uploaded Supporting Information files, but you have not included a list of legends. Please add a full list of legends for your Supporting Information files after the references list.

4. In the online submission form, you indicated that "Due to the sensitive nature of the data and to protect the confidentiality and anonymity of study participants, interview transcripts are not publicly available. Anonymised data summaries may be made available from the corresponding author upon reasonable request.".

All PLOS journals now require all data underlying the findings described in their manuscript to be freely available to other researchers, either

1. In a public repository,

2. Within the manuscript itself, or

3. Uploaded as supplementary information.

This policy applies to all data except where public deposition would breach compliance with the protocol approved by your research ethics board. If your data cannot be made publicly available for ethical or legal reasons (e.g., public availability would compromise patient privacy), please explain your reasons by return email and your exemption request will be escalated to the editor for approval. Your exemption request will be handled independently and will not hold up the peer review process, but will need to be resolved should your manuscript be accepted for publication. One of the Editorial team will then be in touch if there are any issues.

If the reviewer comments include a recommendation to cite specific previously published works, please review and evaluate these publications to determine whether they are relevant and should be cited. There is no requirement to cite these works unless the editor has indicated otherwise.

Additional Editor Comments (if provided):

[Note: HTML markup is below. Please do not edit.]

Reviewers' comments:

Reviewer's Responses to Questions

**Comments to the Author**

1. Does this manuscript meet PLOS Global Public Health’s publication criteria?

Reviewer #1: Yes

Reviewer #2: Yes

**********

2. Has the statistical analysis been performed appropriately and rigorously?

Reviewer #1: N/A

Reviewer #2: N/A

**********

3. Have the authors made all data underlying the findings in their manuscript fully available (please refer to the Data Availability Statement at the start of the manuscript PDF file)?

The PLOS Data policy

Reviewer #1: Yes

Reviewer #2: No

**********

4. Is the manuscript presented in an intelligible fashion and written in standard English?

Reviewer #1: Yes

Reviewer #2: Yes

**********

Reviewer #1: The paper uses standard scientific methods and the paper is robust methodologically. It is relatively weak on the novelty front. I have a few suggestions.

1. Gutka and smokeless tobacco: Gutka is a specific type of smokeless tobacco (tobacco, lime, areca nut and other ingredients). The authors acknowledge this. However, while presenting the quotations from the participants, there seem to be an overlap between smokeless tobacco and Gutka. This is important from policy perspective as Gutka may have been banned at the provincial level (or possibly at the federal level in Pakistan) but raw tobacco may not be covered by the ban. Authors may clarify on the ban status of various smokeless tobacco products. The authors may also clarify about the overlap between the two. As pointed out in the data, users shift from one product to another product based on affordability, availability, experimentation, etc. Since the paper is about Gutka, it is important to be elaborate on this.

2. shopkeepers as users or as suppliers: In South Asia, there is anecdotal evidence that tobacco vendors if targeted by law enforcement agencies, the selling points shift to other shops including cloth shops, which makes law enforcement extremely difficult. It is not clear from the paper whether the participants were also selling tobacco.

3. In South Asia, there have been manufacturing industries which sell paan masala without tobacco. By this tactic, they are able to legally sell the product and the point-of-purchase, these are often sold along with loose tobacco. Were such dynamics also reported by the participants. If not, is there any evidence in this regard from the preexisting literature?

4. Choice of cloth shopkeepers: The authors have used therms such as commercial retail, informal occupational environments but have selected only cloth shopkeepers. What are the underlying reasons? Why did the authors not select any other retailers from Karachi markets? What do the authors mean by informal occupational environment (informal sector or informal environment which exists in every occupational/organisational setting irrespective of whether the setting is formal or informal).

5. Lines 167-169:The authors report having excluded persons with history of cancer and other major illnesses. I can understand the rationale as their views are likely to be far different than the current users. However, I do not agree with the statement that this group will have bias in reporting health outcomes. They already have encountered a Non-communicable disease and I feel that they would simply report it and may (or may not) attribute it to tobacco use but will not have bias in reporting the outcome. Please re-frame the sentence or clarify.

6. Table reflects the quotations presented except 1.2 where divine will has been mentioned. I could not find quotation indicating that.

Reviewer #2: This manuscript presents the results of original research in a well-structured and well written manner.

Aim/Objectives:

The aim and objectives need to be reviewed and fine-tuned, with the objectives leading to the aim.

Methods:

Which approach was used for this qualitative work? Phenomenology, Grounded theory or any other?

Please provide details of how the sample size of 25 male cloth shopkeepers was constituted.

Exclusion of female participants need to be justified.

There is need for data analysis to be undertaken by two independent persons.

It is noted that the research work received ethical clearance

It is also noted that authors included a paragraph on reflexivity.

On the other hand, authors need to adhere to the relevant guidelines for qualitative studies.

Results:

There is need to remove the name of the market where the participant is based so as to preserve the anonymity of participants and avoid unnecessary identification.

Conclusions are derived from the data.

**********

what does this mean?). If published, this will include your full peer review and any attached files.

**Do you want your identity to be public for this peer review?**  If you choose “no”, your identity will remain anonymous but your review may still be made public.

For information about this choice, including consent withdrawal, please see our Privacy Policy

Reviewer #1: No

Reviewer #2: **Yes:**  Marie CHAN SUN

**********

[NOTE: If reviewer comments were submitted as an attachment file, they will be attached to this email and accessible via the submission site. Please log into your account, locate the manuscript record, and check for the action link "View Attachments". If this link does not appear, there are no attachment files.]

# Author comment: Author response to Decision Letter 1

**DOI:** 10.1371/journal.pgph.0007567.r002

2 Jul 2026

Attachment Submitted filename: Gutka_Response Sheet_30062026.docx

# Peer review: Decision Letter 1

**DOI:** 10.1371/journal.pgph.0007567.r003

**Authors:** Emma Campbell

19 Aug 2026

PGPH-D-26-01732R1

Normalised, Accessible, and Addictive: Socioecological Drivers of Gutka Consumption Among Urban Cloth Shopkeepers in Karachi, Pakistan

PLOS Global Public Health

Dear Dr. Sameen,

Thank you for submitting your manuscript to PLOS Global Public Health. After careful consideration, we feel that it has merit but does not fully meet PLOS Global Public Health’s publication criteria as it currently stands. Therefore, we invite you to submit a revised version of the manuscript that addresses the points raised during the review process.

The revised manuscript has been assessed and the reviewer comments can be found below. Concerns regarding the methodological reporting are still present, please review the comments from the reviewers and make the appropriate revisions to address the concerns raised.

Please submit your revised manuscript by Oct 18 2026 11:59PM. If you will need more time than this to complete your revisions, please reply to this message or contact the journal office at globalpubhealth@plos.org. When you're ready to submit your revision, log on to https://www.editorialmanager.com/pgph/ and select the 'Submissions Needing Revision' folder to locate your manuscript file.

Please include the following items when submitting your revised manuscript:

- A letter that responds to each point raised by the editor and reviewer(s). You should upload this letter as a separate file labeled 'Response to Reviewers'.
- A marked-up copy of your manuscript that highlights changes made to the original version. You should upload this as a separate file labeled 'Revised Manuscript with Track Changes'.
- An unmarked version of your revised paper without tracked changes. You should upload this as a separate file labeled 'Manuscript'.

Guidelines for resubmitting your figure files are available below the reviewer comments at the end of this letter.

As the corresponding author, your ORCID iD is verified in the submission system and will appear in the published article. PLOS supports the use of ORCID, and we encourage all coauthors to register for an ORCID iD and use it as well. Please encourage your coauthors to verify their ORCID iD within the submission system before final acceptance, as unverified ORCID iDs will not appear in the published article. *Only*  the individual author can complete the verification step; PLOS staff *cannot*  verify ORCID iDs on behalf of authors.

We look forward to receiving your revised manuscript.

Kind regards,

Emma Campbell, Ph.D

Staff Editor

PLOS Global Public Health

Journal Requirements:

If the reviewer comments include a recommendation to cite specific previously published works, please review and evaluate these publications to determine whether they are relevant and should be cited. There is no requirement to cite these works unless the editor has indicated otherwise.

Additional Editor Comments (if provided):

[Note: HTML markup is below. Please do not edit.]

Reviewers' comments:

Reviewer's Responses to Questions

**Comments to the Author**

Reviewer #1: All comments have been addressed

Reviewer #3: All comments have been addressed

**********

publication criteria?

Reviewer #1: Yes

Reviewer #3: Partly

**********

3. Has the statistical analysis been performed appropriately and rigorously?

Reviewer #1: N/A

Reviewer #3: No

**********

4. Have the authors made all data underlying the findings in their manuscript fully available (please refer to the Data Availability Statement at the start of the manuscript PDF file)?

The PLOS Data policy

Reviewer #1: Yes

Reviewer #3: Yes

**********

5. Is the manuscript presented in an intelligible fashion and written in standard English?

Reviewer #1: Yes

Reviewer #3: Yes

**********

**Review Comments to the Author**

Reviewer #1: The comments have been addressed.

Reviewer #3: The manuscript addresses an important and understudied public health issue and is generally well written, methodologically sound, and grounded in a robust socioecological framework. The findings provide valuable insights into the occupational, social, and structural determinants of gutka consumption among cloth shopkeepers in Karachi. Overall, the study offers meaningful policy implications and makes a worthwhile contribution to the smokeless tobacco literature.

Major Suggestions

1. Enhance Methodological Transparency While the study is described as a qualitative exploratory design using thematic analysis, the specific qualitative orientation remains unclear. Explicitly stating the epistemological stance and methodological approach (e.g., interpretivist paradigm and qualitative descriptive approach) would strengthen methodological rigor and improve alignment with qualitative reporting standards. [PGPH-D-26-...732_R1 (1) | PDF]

2. Provide a More Detailed Account of Data Saturation The manuscript indicates that thematic saturation was achieved but does not explain how saturation was determined. Clarification regarding the type of saturation reached (code or meaning saturation), the stage at which it occurred, and the process used to document it would improve methodological credibility.

3. Expand Participant Characteristics The participant profile is currently limited. Inclusion of additional contextual characteristics such as educational status, income category, duration of gutka use, average daily consumption, and type of gutka consumed (commercial versus homemade) would facilitate richer interpretation of the findings.

4. Strengthen Integration of the Socioecological Framework The framework is effectively applied in presenting findings; however, the discussion could better articulate interactions across levels. Greater emphasis on how affordability, workplace culture, social norms, accessibility, and policy failures interact to sustain gutka use would enhance the theoretical contribution of the study.

5. Further Emphasize Occupational Health Implications One of the manuscript’s most novel contributions is its focus on an occupationally vulnerable population. The discussion would benefit from deeper consideration of workplace-based tobacco control strategies, occupational stress, health promotion programs, and market-specific cessation interventions.

6. Expand Discussion on Product Substitution Participants reported shifting to naswar and homemade preparations when commercial gutka became less accessible or more expensive. This finding has important regulatory implications and warrants a more focused discussion on product substitution, harm reduction misconceptions, and challenges associated with regulating homemade products.

Minor Suggestions

7. Refine Aim and Objectives The distinction between the study aim and objectives could be improved to ensure clearer alignment and reduce overlap. Objectives should directly reflect the socioecological determinants, perceived health effects, and views on existing control measures.

8. Strengthen the Conceptual Figure The current socioecological framework is informative but primarily descriptive. A more explanatory model showing pathways such as early initiation leading to addiction, peer influence fostering normalization, and enforcement failures sustaining availability would enhance the manuscript’s conceptual contribution.

9. Discuss Transferability The limitations section could better address the applicability of findings to other occupational groups such as vendors, retail workers, informal-sector employees, and market traders in similar South Asian contexts.

10. Provide More Specific Practice and Policy Recommendations The conclusion would be strengthened by outlining actionable recommendations, such as workplace-based cessation services, mobile tobacco cessation clinics, peer-support programs, tobacco-free market campaigns, enhanced enforcement against youth access, and regulation of ingredients used in homemade gutka production.

Overall Recommendation

Minor Revision

**********

what does this mean?). If published, this will include your full peer review and any attached files.

**Do you want your identity to be public for this peer review?**  If you choose “no”, your identity will remain anonymous but your review may still be made public.

For information about this choice, including consent withdrawal, please see our Privacy Policy

Reviewer #1: No

Reviewer #3: No

**********

[NOTE: If reviewer comments were submitted as an attachment file, they will be attached to this email and accessible via the submission site. Please log into your account, locate the manuscript record, and check for the action link "View Attachments". If this link does not appear, there are no attachment files.]

# Author comment: Author response to Decision Letter 2

**DOI:** 10.1371/journal.pgph.0007567.r004

31 Aug 2026

Attachment Submitted filename: Gutka_Response Sheet_28082026.docx

# Peer review: Decision Letter 2

**DOI:** 10.1371/journal.pgph.0007567.r005

**Authors:** Julia Robinson

19 Oct 2026

Normalised, Accessible, and Addictive: Socioecological Drivers of Gutka Consumption Among Urban Cloth Shopkeepers in Karachi, Pakistan

PGPH-D-26-01732R2

Dear Dr. Sameen,

We are pleased to inform you that your manuscript 'Normalised, Accessible, and Addictive: Socioecological Drivers of Gutka Consumption Among Urban Cloth Shopkeepers in Karachi, Pakistan' has been provisionally accepted for publication in PLOS Global Public Health.

Before your manuscript can be formally accepted you will need to complete some formatting changes, which you will receive in a follow up email. A member of our team will be in touch with a set of requests.

Please note that your manuscript will not be scheduled for publication until you have made the required changes, so a swift response is appreciated.

IMPORTANT: The editorial review process is now complete. PLOS will only permit corrections to spelling, formatting or significant scientific errors from this point onwards. Requests for major changes, or any which affect the scientific understanding of your work, will cause delays to the publication date of your manuscript.

If your institution or institutions have a press office, please notify them about your upcoming paper to help maximize its impact. If they'll be preparing press materials, please inform our press team as soon as possible -- no later than 48 hours after receiving the formal acceptance. Your manuscript will remain under strict press embargo until 2 pm Eastern Time on the date of publication. For more information, please contact globalpubhealth@plos.org.

Thank you again for supporting Open Access publishing; we are looking forward to publishing your work in PLOS Global Public Health.

Best regards,

Julia Robinson

Executive Editor

PLOS Global Public Health

***********************************************************

Reviewer Comments (if any, and for reference):

Reviewer's Responses to Questions

**Comments to the Author**

Reviewer #1: All comments have been addressed

**********

publication criteria?

Reviewer #1: Yes

**********

3. Has the statistical analysis been performed appropriately and rigorously?

Reviewer #1: Yes

**********

4. Have the authors made all data underlying the findings in their manuscript fully available (please refer to the Data Availability Statement at the start of the manuscript PDF file)?

The PLOS Data policy

Reviewer #1: Yes

**********

5. Is the manuscript presented in an intelligible fashion and written in standard English?

Reviewer #1: Yes

**********

Reviewer #1: All comments have been addressed.

**********

what does this mean?). If published, this will include your full peer review and any attached files.

**Do you want your identity to be public for this peer review?**  If you choose “no”, your identity will remain anonymous but your review may still be made public.

For information about this choice, including consent withdrawal, please see our Privacy Policy

Reviewer #1: No

**********
